# Preoperative Neutrophil-to-Lymphocyte Ratio Plus Platelet-to-Lymphocyte Ratio Predicts the Outcomes after Curative Resection for Hepatocellular Carcinoma

**DOI:** 10.1155/2019/4239463

**Published:** 2019-04-02

**Authors:** T. Kabir, M. Ye, N. A. Mohd Noor, W. Woon, S. P. Junnarkar, V. G. Shelat

**Affiliations:** Department of General Surgery, Tan Tock Seng Hospital, Singapore

## Abstract

**Background:**

In recent years, inflammation-based scoring systems have been reported to predict survival in Hepatocellular Carcinoma (HCC). The aim of our study was to validate combined preoperative Neutrophil-to-Lymphocyte ratio (NLR)-Platelet-to-Lymphocyte ratio (PLR) in predicting overall survival (OS) and recurrence free survival (RFS) in patients who underwent curative resection for HCC.

**Methods:**

We conducted a retrospective study of HCC patients underwent liver resection with curative intent from January 2010 to December 2013. Receiver-operating characteristic (ROC) curve analysis was used to determine the optimal cut-off values for NLR and PLR. Patients with both NLR and PLR elevated were allocated a score of 2; patients showing one or neither of these indices elevated were accorded a score of 1 or 0, respectively.

**Results:**

132 patients with a median age of 66 years (range 18-87) underwent curative resection for HCC. Overall morbidity was 30.3%, 30-day mortality was 2.3%, and 90-day mortality was 6.8%. At a median follow-up of 24 months (range 1-88), 25% patients died, and 40.9% had recurrence. On multivariate analysis, elevated preoperative NLR-PLR was predictive of both OS (HR 2.496; CI 1.156-5.389;* p*=0.020) and RFS (HR 1.917; CI 1.161-3.166;* p*=0.011). The 5-year OS was 76% for NLR-PLR=0 group, 21.7% for the NLR-PLR=1 group, and 61.1% for the NLR-PLR=2 group, respectively. The 5-year RFS was 39.3% for the NLR-PLR=0 group, 18.4% for the NLR-PLR=1 group, and 21.1% for the NLR-PLR=2 group, respectively.

**Conclusion:**

The preoperative NLR-PLR is predictive of both OS and RFS in patients with HCC undergoing curative liver resection.

## 1. Introduction

Hepatocellular carcinoma (HCC) poses a significant health problem globally, as it is the second leading cause of cancer-related death and the sixth most common malignancy worldwide [1, 2]. Prognostication is difficult as survival depends not only on the tumour burden, but also on the degree of underlying liver dysfunction [3]. In the Asia-Pacific region which accounts for almost 75% of cases [4], partial liver resection is still considered the mainstay of curative therapy. Unfortunately, recurrence rates may be as high as 50-70% [5]. It is thus imperative that we select patients for surgery appropriately, in order to avoid futile interventions that do not significantly improve outcomes.

In recent years, there is increasing evidence that a systemic inflammatory response is associated with poor survival in patients with various malignancies, including HCC [6]. Several inflammation-based prognostic scores have been studied, such as the Glasgow Prognostic Score (GPS) [7], Neutrophil-to-Lymphocyte ratio (NLR) [8], Platelet-to-Lymphocyte ratio (PLR) [6], and Prognostic Nutritional Index (PNI) [9]. In a prospective study of 113 patients who underwent curative resection for HCC, Yamamura et al. concluded that NLR was an independent predictor of recurrence free survival (RFS) [8]. However, Huang suggested that preoperative GPS was superior in predicting survival outcomes after hepatectomy [9]. Others have reported that preoperative PLR also predicts overall survival (OS) after hepatectomy [10]. As such, there is no consensus yet as to which is the best scoring system.

Some authors have shown that a combination of scores may be more helpful in prognosticating outcomes in patients with HCC [11, 12]. The NLR and PLR are two of the most widely used scores in HCC which have been studied extensively on their own, as well as in addition with other scoring systems. To the best of our knowledge, these scores have not yet been evaluated in combination with each other in the preoperative setting of HCC. The aim of our present study was to investigate whether preoperative NLR plus PLR may predict overall survival (OS) and recurrence free survival (RFS) in patients who underwent curative resection for HCC.

## 2. Materials and Methods

We conducted a retrospective study of HCC patients who underwent liver resection with curative intent from January 2010 to December 2013. The diagnosis of HCC was established by postoperative histology. Baseline demographic profile, clinical data, and laboratory parameters were retrieved from electronic medical records. Blood samples were drawn from patients prior to surgery as part of the routine preoperative workup. Complete blood count, serum albumin, liver function, renal function, hepatitis B and C status, serum alpha-fetoprotein (AFP), Child-Pugh score, and indocyanine-green retention rate at 15 minutes (ICG15) were recorded.

Neutrophil-to-Lymphocyte ratio (NLR) was calculated as absolute neutrophil count (number of neutrophils/*μ*L) divided by absolute lymphocyte count (number of lymphocytes/*μ*L). The cut-off values were 2.3 and 3.1 based on the receiver-operating characteristic (ROC) curve for OS and RFS respectively, and hence an average value of 2.7 was chosen.

Platelet-to-Lymphocyte ratio (PLR) was calculated as absolute platelet count (number of platelets/*μ*L) divided by absolute lymphocyte count (number of lymphocytes/*μ*L). The cut-off values were 176 and 133 based on the ROC curves for OS and RFS, respectively; similarly the average value of 155 was chosen.

A new inflammation-based score, the NLR-PLR score, was generated by combining the NLR score with the PLR score. Patients with both NLR and PLR elevated were allocated a score of 2, patients with either NLR or PLR elevated were allocated a score of 1, and patients with both NLR and PLR below the cut-off values were accorded a score of 0.

Cardiopulmonary exercise test was performed selectively at the discretion of the operating surgeon. Patients with anaerobic threshold of <11ml/min/kg were deemed to be at high cardiovascular risk for major surgery and thus were considered for nonsurgical treatment modalities [13]. All patients who underwent surgery showed no signs of systemic inflammation or infection at the time of surgery. Indications for hepatectomy and extent of hepatic resection were based on the size, number, and location of tumours; liver function as determined by blood tests, Child-Pugh score, and indocyanine-green (ICG) clearance test. We consider an ICG retention value of >15% at 15 minutes as a cut-off for major liver resection. Computerized tomography (CT) liver volumetry was calculated for selected patients planned for major liver resection and consensus guidelines were used to identify those with adequate future liver remnant (FLR) volumes [14]. Nomenclature of resection was defined according to the Brisbane 2000 classification [15]. Major hepatectomy was defined as the resection of three or more segments and minor hepatectomy defined as the resection of fewer than three segments [16]. Histology reports were reviewed for resection margins. All patients had resection with intent of cure and R0 resection was defined as histological negative margins.

Intraoperative data such as the estimated blood loss (EBL) and surgical time were recorded. Tumour specific characteristics such as size, number of lesions, and margin status were determined based on histopathologic reports. Postoperative morbidity, 30-day mortality, and 90-day mortality were reported. Length of stay was calculated from the date of surgery to date of discharge, inclusive of both dates. Upon discharge, patients were followed with physical examination, liver function test, AFP, and multiphasic CT scan according to local protocol [17]. Site of recurrence was determined from clinical records and imaging. RFS was calculated from the date of surgery to the date of recurrence and was censored at the last follow-up or at the time of death if the patients remained tumour free at that time. Overall survival (OS) was calculated from the time of surgery to the date of cancer-related death and was censored at last follow-up or at death not related to cancer.

All patients for liver resection received calf compressor devices, Bair hugger, low central venous pressure anesthesia, surgical infection prophylaxis, and intraoperative glycaemia monitoring according to our institutional policy [17, 18]. For open liver resections, a reverse “L” incision was made. Liver mobilization and portal sling were routinely performed. Pringle maneuver was performed selectively at the discretion of the operating surgeon and done in cycles of “10 minutes on” and “5 minutes off”. Intraoperative ultrasound was performed to tattoo the resection margin and delineate the relation of major blood vessels to the tumour and resection plane. Parenchymal transection was achieved using Sonosurg™ (Olympus, Tokyo, Japan) and LigaSure™ (Covidien, Minneapolis, USA) with dolphin tip. Tubular structures of >3mm were ligated or clipped as necessary. Major pedicles were stapled with vascular stapling device. After transection, the raw surface of the liver was covered with Tachosil™ (Baxter, Illinois, US) or EVICEL™ glue (Ethicon US, Cincinnati, OH) at the discretion of surgeon. We routinely place drains after major hepatectomy and selectively after minor hepatectomy. Drains were removed when the output was <50ml/24 hr or at the discretion of surgeon. Postoperatively, all patients were monitored in the surgical high dependency ward for 24-48 hours and managed under a standardized liver resection care pathway.

Our unit started performing laparoscopic liver resection since July 2006 and since then, the technique has been refined and criteria have been expanded. The technique is similar to open surgery except portal sling which is not routinely achieved. An intraoperative ultrasound is routinely done akin to open surgery. In instances of major intraoperative bleeding we achieve hemostasis with intracorporeal suturing assisted by transiently elevated intraperitoneal pressures, and open conversion is promptly performed if this fails. Parenchymal transection is carried out using LigaSure™ (Covidien, Minneapolis, USA) or Thunderbeat™ (Olympus, Tokyo, Japan) in laparoscopic liver resection assisted with liberal usage of stapling devices. In wedge resections, we selectively place stay sutures to help traction-retraction. In major resections, we prefer inflow control prior to parenchymal transection.

## 3. Statistical Analysis

Continuous variables were presented as median and range and compared using the Mann-Whitney U test. Categorical variables were compared using the Chi square test or Fischer's exact test. Cox proportional hazards model was used for univariate and multivariate analysis. Factors that were found to be significant predictors for RFS and OS in univariate analysis were entered into a multivariate analysis. The criterion for statistical significance was set at *α* of 0.01, and all p values were based on two-sided tests. Cumulative OS and RFS curves were analyzed using the Kaplan–Meier method and compared using the log-rank test.

All the statistical analyses were performed using SPSS statistical software package, version 19.0 (SPSS, Inc., Chicago, US).

## 4. Results

153 patients underwent liver resection over 4-year period from January 2010 to December 2013. 21 patients were excluded as liver resection was performed for symptomatic benign lesions (n=6), colorectal liver metastases (n=12), cholangiocarcinoma (n=2), and breast cancer liver metastases (n=1). The final analysis is comprised of 132 patients.  Table 1 provides a summary of the clinicopathological characteristics of the patients. Patients were mostly males (87.9%), with a mean age of 65.2 ± 10.2 years. 56.8% were Hepatitis B carriers. Most patients were Child-Pugh Class A, with only 9.8% being Child-Pugh Class B. 13.6% of patients had elevated AFP >400ug/L.

Major liver resections were performed in 46.2% of patients, and 61.4% had estimated blood loss (EBL) >500mls. Most patients had R0 resection, with 9.8% having R1 resection.

The overall morbidity rate was 30.3%, of which pneumonia (16.7%) and liver failure (8.3%) were the commonest causes. 30-day mortality was 2.3% and 90-day mortality was 6.8%. Mean length of stay was 14.0 ± 19.2 days. Median follow-up duration was 24 months (range 1-88 months). At the time of analysis, 25% of patients died and 40.9% had recurrence. The median OS was 24 months (range 1-88 months) and median RFS was 17.5 months (range 1-84 months).

The results of the Cox regression hazards model for predictors of OS are shown in Table 2. On univariate analysis, operation time >350min, EBL>500ml, tumour size >5cm, R1 resection, elevated PLR, elevated NLR, and elevated NLR-PLR were potential risk factors for OS. However on multivariate analysis, only EBL>500ml (hazard ratio (HR) 5.29; 95% CI 1.577-17.712;* p*=0.007), R1 resection (HR 3.112; 95% CI 1.251-7.443;* p*=0.015), and elevated NLR-PLR (HR 2.496; CI 1.156-5.389;* p*=0.020) were independently associated with unfavourable OS.

The results of the Cox regression hazards model for predictors of RFS are shown in Table 3. On univariate analysis, EBL>500ml, R1 resection, elevated NLR, elevated PLR, and elevated NLR-PLR were potential risk factors for recurrence after curative liver resection. However on multivariate analysis, only EBL>500ml (HR 1.781; CI 1.017-3.120;* p*=0.043), R1 resection (HR 2.34; HR 1.093-5.010;* p*=0.029), and elevated NLR-PLR (HR 1.917; CI 1.161-3.166;* p*=0.011) were independently associated with postoperative recurrence.

We subsequently compared the characteristics of patients with preoperative NLR-PLR scores of 0, 1, and 2, as shown in Table 4. There were 64 patients in the NLR-PLR 0 group, 32 patients in the NLR-PLR 1 group, and 36 patients in the NLR-PLR 2 group. The NLR-PLR 2 group had a larger proportion of patients with Child-Pugh Class B, major hepatectomy, and tumour size >5cm (p<0.01), while the NLR-PLR 0 group had more patients with ICG>15 (p<0.01). There were otherwise no significant differences.

**Figure 1:**
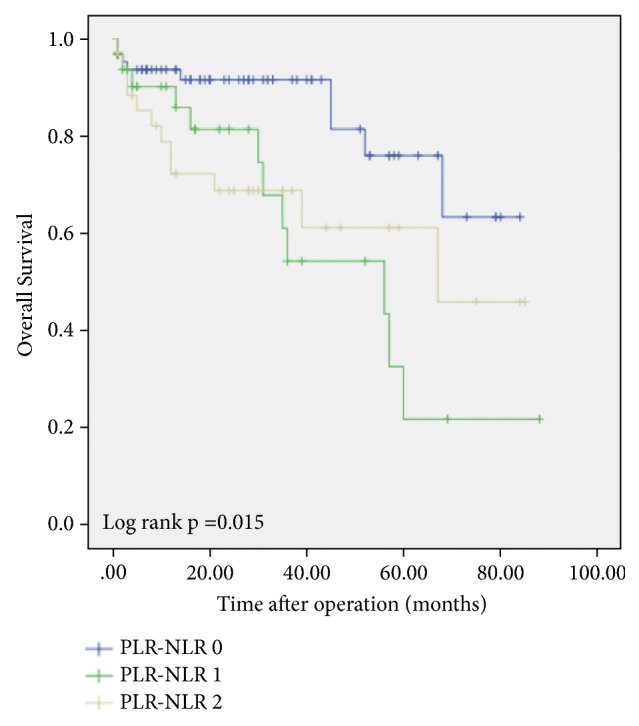
Kaplan–Meier survival plots comparing overall survival for patients with preoperative NLR-PLR score of 0, 1, and 2.

**Figure 2:**
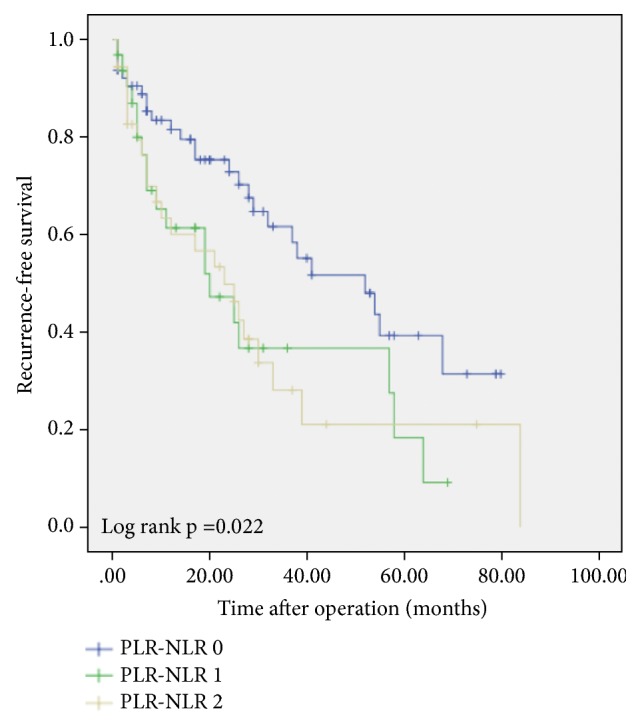
Kaplan–Meier survival plots comparing recurrence free survival for patients with preoperative NLR-PLR score of 0, 1, and 2.

Figures 1 and 2 exhibit the Kaplan–Meier survival curves for OS and RFS for patients across the different NLR-PLR scores.

The 1-, 3-, and 5-year OS were 93.6%, 91.6%, and 76% for NLR-PLR=0 group, 90.2%, 54.2%, and 21.7% for the NLR-PLR=1 group and 78.8%, 68.8%, and 61.1% for the NLR-PLR=2 group, respectively.

The 1-, 3-, and 5-year RFS were 81.6%, 61.7%, and 39.3% for the NLR-PLR=0 group, 61.4%, 36.7%, and 18.4% for the NLR-PLR=1 group and 60.1%, 28.1%, and 21.1% for the NLR-PLR=2 group, respectively.

## 5. Discussion

Owing to the shortage of organs for transplantation, partial liver resection (LR) is still the treatment of choice for patients with resectable HCC, particularly in Asia [19]. Surgery, however, is not without its attendant risks. Patients often have impaired liver function due to chronic hepatitis or cirrhosis, and morbidity rates after surgery may be as high as up to 47.7% [20]. These include bile leaks, liver failure, renal failure, and organ space infections. In addition, 5-year recurrence rates may be as high as 60-70%, and 10-year survival rates are dismal at only 7-14% [21]. In order to avoid “futile” surgery, it is crucial to identify pretreatment factors that allow us to select patients appropriately for hepatectomy based on their individual risk-benefit ratios. Preoperative inflammation-based scores are easy to calculate from routine biochemical tests, inexpensive, and have been shown to prognosticate outcomes following surgery in various malignancies, including HCC. Our study is the first to confirm that an elevated preoperative combined NLR-PLR score is predictive of both OS and DFS following curative LR for HCC. In addition to this, we found that increased EBL [22] as well as R1 resection [23] were also independently associated with poor survival after liver resection, which is concordant with other studies [24].

To date, several studies have evaluated the role of pretreatment PLR in prognosticating outcome in HCC. A recent meta-analysis of 2315 patients who underwent either surgery or transarterial chemoembolization (TACE) for HCC found that elevated PLR was significantly associated with worse OS compared to the low PLR group [HR =1.60, 95% CI = 1.23-2.08,* p*=0.0005] [25]. Another meta-analysis comprising of 2449 HCC patients across different BCLC stages similarly showed that high pretreatment PLR correlated with unfavourable OS (HR = 1.73; 95% CI: 1.46, 2.04; P < 0.00001) and DFS (HR = 1.30; 95% CI: (1.06, 1.60); P = 0.01) [26]. Focusing only on patients with early-stage HCC amenable to resection, a comparison of five well-known inflammation-based scores confirmed that preoperative PLR was an independent predictor of recurrence beyond the Milan criteria [27]. In another retrospective review, 778 patients were divided into 5 quintiles based on their preoperative PLR scores [10]. PLR was shown to be an independent risk factor for OS (p=0.003), and in a subgroup analysis, PLR quintiles were significantly associated with poor OS in HBsAg positive and cirrhotic patients.

NLR has also been studied extensively in HCC. In various reports, high NLR has been shown to be a predictor of poor survival after radio-frequency ablation [28], TACE [29], and liver transplantation for HCC [30]. A large meta-analysis of 17 studies was recently published, which analyzed both retrospective and prospective studies of patients who only underwent curative surgery for HCC [31]. The results showed that elevated preoperative NLR was predictive of the OS (HR 1.52; 95% CI 1.37–1.69) and RFS (HR 1.64; 95% CI 1.44–1.87) as well as disease-free survival (DFS) (HR 1.50; 95% CI 1.35–1.67) of HCC. In addition, NLR was also associated with large size of tumour and vascular invasion as well as Hep B positivity.

Due to the lack of optimal cut-off values in inflammation-based scores such as NLR and PLR, a range of values has been used in over the years with varying outcomes [25, 31]. Hence, we hypothesised that a combination of scores may be more accurately reflective of ongoing chronic inflammatory states and outcomes following hepatectomy. A combined pretreatment NLR-PNI score has been shown to be superior in predicting OS for patients with unresectable HCC undergoing TACE [11]. In patients treated with surgical resection alone, NLR combined with aspartate aminotransferase/platelet count ratio index (APRI) was found to be more sensitive in predicting survival than either measure alone [12].

To date, only one study has investigated the role of NLR-PLR score in prognosticating HCC outcomes [13]. Li et al. analyzed the postoperative NLR-PLR scores recorded within one month after liver resection and concluded that it was predictive of both OS (HR 2.894, 95% CI 1.992-4.2, p<0.01) and RFS (HR 1.711, 95% CI 1.323-2.265, p<0.01). Unlike our present study, their study utilised postresection scores. In their patient cohort, pretreatment NLR and PLR scores were not individually predictive of outcomes, and neither was the combined preoperative NLR-PLR score. The authors suggested that the stress induced by surgery itself may contribute to the overall systemic inflammatory state which in turn affects survival outcomes; hence they chose to focus on the postoperative blood markers. However, we are concerned that acute postoperative infections such as intra-abdominal collections, bile leak, or even nosocomial infections may affect serum neutrophil, platelet, and lymphocyte levels and confound results. In contrast, presurgery patients are usually free of acute infective or inflammatory conditions. Hence the cell counts from blood drawn at that time are likely to be a more accurate reflection of the ongoing cancer-induced chronic inflammatory state. Furthermore, Li's study was restricted to patients with only HBV-related HCC, whereas our study also included patients with Hepatitis C, as well as patients without hepatitis.

A number of theories have been proposed as to why elevated NLR and PLR are negatively associated with survival and recurrence. High NLR and PLR reflect neutrophilia and thrombocytosis due to the presence of tumour-associated macrophages secreting inflammatory cytokines such as interleukin-6 (IL-6) and IL-17 [32, 33]. The neutrophils and platelets may be involved in tumorigenesis and angiogenesis [34–36], promoting motility of cancer cells [37], expression of matrix metalloproteases [38], and promoting tumour invasion and metastasis. Activated platelets assist tumour cells to evade immune elimination by promoting their arrest at the endothelium, thus enhancing the establishment of secondary lesions [39, 40]. In contrast, lymphocytes play a significant role in cancer immune-surveillance. Lymphocyte depletion reflects an impaired T lymphocyte-mediated antitumor response [41], and lymphocytopenia has been reported to be associated with poorer survival outcomes in patients with pancreatic cancer and other gastrointestinal malignancies [42].

It is also worthwhile to note in our study that the NLR-PLR 2 group had a significantly higher proportion of patients with tumour size >5cm (p<0.01) and Child-Pugh Class B (p<0.01), which are well-known risk factors for poor outcomes following LR [24]. Interestingly, multivariate analysis did not reveal tumour size and Child-Pugh Class B to be independent predictors of survival in our experience. Perhaps the elevated NLR-PLR scores hint to a more florid ongoing chronic inflammatory state in patients with larger tumours and Child-Pugh Class B liver disease.

Additionally, we discovered that the OS and RFS for our NLR-PLR 1 group was slightly worse than the NLR-PLR 2 group as reflected in the KM curves, which is counter-intuitive. There is statistically no significant difference in OS and RFS between these 2 groups. We attribute this to the small numbers of patients in each group (32 and 36 resp.).

This study has several limitations. Firstly, it is a retrospective single institution study with a relatively small sample; hence it is prone to selection bias. Secondly, there was no established optimal time to draw blood samples from patients preoperatively. In our study, we collected the blood when our patients came for their preoperative anaesthetic workup, which was typically within 10 days of the surgery. Thirdly, there were no standardized optimal cut-offs for NLR and PLR in the literature and thresholds used in this study were from calculations derived from our own cohort and thus have not been validated in an independent cohort. Fourthly, the reference ranges for each parameter are affected by the waiting period to analysis and this can introduce bias [43]. Finally, weight loss, unidentified sepsis, instrumental error, and unknown tumour related haemorrhage may alter the markers and ideally samples should be analyzed more than once to avoid random errors [44]. This can only be done in a prospective study.

## 6. Conclusion

In conclusion, this study demonstrated that an elevated preoperative NLR-PLR score as well as higher blood loss and R1 margins predicted a poorer prognosis in patients who underwent curative surgery for HCC. This information may potentially be very useful to surgeons in the selection and counseling of patients for hepatectomy.

## Figures and Tables

**Table 1:** Clinicopathological characteristics of patients undergoing curative hepatectomy for HCC.

Clinical variables	Overall (n=132) (%)
*Patient factors*	
Male (n, %)	116 (87.9%)
Age (years; mean ± SD)	65.2 ± 10.2
Hepatitis B (n, %)	75 (56.8%)
Child-Pugh Class B (n, %)	13 (9.8%)

*Laboratory data*	
ICG>15 (n, %)	30 (22.7%)
Creatinine (mean ± SD)	98±91

*Surgical factors*	
Major hepatectomy (n, %)	61 (46.2%)
Operation time >350min (n, %)	43 (32.6%)
EBL >500ml (n, %)	81 (61.4%)

*Tumor factors*	
AFP >400ug/L (n, %)	18 (13.6%)
Tumor size >5cm (n, %)	60 (45.4%)
Multiple tumors (n, %)	30 (22.7%)
R1 resection (n, %)	13 (9.8%)

*Postoperative factors*	
Wound infection	6 (4.5%)
Pneumonia	22 (16.7%)
UTI	7 (5.3%)
Bile leak	2 (1.5%)
Intra-abdominal collection	4 (3.0%)
Liver failure	11 (8.3%)

*Follow-up*	
30-day mortality	3 (2.3%)
LOS (days, mean ± SD)	14.0 ± 19.2
OS (months, median [IQR])	24(1-88)
RFS (months, median [IQR])	17(1-84)

ICG: indocyanine green; EBL: estimated blood loss; AFP: a-fetoprotein; UTI: urinary tract infection; LOS: length of stay; OS: overall survival; RFS: recurrence free survival; IQR: interquartile range; SD: standard derivation.

**Table 2:** Univariate and multivariate analysis of prognostic factors for overall survival.

Characteristics	Univariate		Multivariate	
	HR (95% CI)	*p* value	HR (95% CI)	*p* value
*Patient factors*				
Age (years)	1.028 [0.994, 1.063]	0.110		
Male sex	1.426 [0.500, 4.069]	0.507		
Hepatitis B	1.462 [0.737,2.898]	0.276		
Child Pugh B	1.806 [0.692, 4.714]	0.227		
*Laboratory data*				
ICG>15	1.353 [0.470, 3.891]	0.575		
Creatinine	0.999 [0.993,1.004]	0.816		
*Surgical factors*				
Major surgery	1.733 [0.859,3.496]	0.125		
Operation time > 350min	2.156 [1.080, 4.301]	**0.029**	1.834 [0.875, 3.842]	0.108
EBL>500ml	5.400 [1.644, 17.739]	**0.005**	5.286 [1.577,17.712]	**0.007**
*Tumor factors*				
AFP>400	1.906 [0.820, 4.431]	0.134		
Tumor size>5cm	2.396 [1.174, 4.888]	**0.016**	1.480 [0.685, 3.197]	0.318
Multiple tumors	1.805 [0.855,3.809]	0.121		
R1 resection	2.817 [1.149, 6.906]	**0.024**	3.112 [1.251, 7.443]	**0.015**
*Inflammatory indices*				
PLR ≥155	2.000 [1.007, 3.973]	**0.033**		
NLR ≥ 2.7	2.175 [1.069, 4.425]	**0.032**		
PLR ≥155 and NLR ≥ 2.7	2.889 [1.342,6.222]	**0.007**	2.496 [1.156,5.389]	**0.020**

HR: hazard ratio, CI: confidence interval, ICG: indocyanine green, EBL: estimated blood loss, AFP: alpha-fetoprotein, NLR: neutrophil-to-lymphocyte ratio, and PLR: platelet-to-lymphocyte ratio.

**Table 3:** Univariate and multivariate analysis of prognostic factors of recurrence free survival.

Characteristics	Univariate		Multivariate	
	HR (95% CI)	*p* value	HR (95% CI)	*p* value
*Patient factors*				
Age (years)	1.012 [0.991, 1.034]	0.266		
Male sex	1.674 [0.796, 3.518]	0.174		
Hepatitis B	1.072 [0.661, 1.737]	0.778		
Child Pugh B	1.692 [0.833, 3.440]	0.146		
*Laboratory data*				
Creatinine	0.998 [0.993, 1.003]	0.461		
ICG>15	1.164 [0.570, 2.373]	0.677		
*Surgical factors*				
Major surgery	1.128 [0.696, 1.828]	0.626		
Operation time > 350min	1.474 [0.890, 2.439]	0.132		
EBL>500ml	1.788 [1.026, 3.113]	**0.040**	1.781 [1.017,3.120]	**0.043**
*Tumor factors*				
AFP >400	1.331 [0.695, 2.549]	0.388		
Size>5cm	1.498 [0.926, 2.423]	0.100	0.909 [0.522,1.581]	0.735
Multiple tumors	1.293 [0.734,2.276]	0.374		
R1 resection	2.492 [1.215, 5.109]	**0.013**	2.340 [1.093,5.010]	**0.029**
*Inflammatory indices*				
PLR ≥155	1.717 [1.047, 2.814]	**0.032**		
NLR ≥ 2.7	1.751 [1.078, 2.845]	**0.032**		
PLR ≥155 and NLR ≥ 2.7	2.115 [1.294,3.455]	**0.003**	1.917 [1.161,3.166]	**0.011**

HR: hazard ratio, CI: confidence interval, ICG: indocyanine green, EBL: estimated blood loss, AFP: alpha-fetoprotein, NLR: neutrophil-to-lymphocyte ratio, and PLR: platelet-to-lymphocyte ratio.

**Table 4:** Clinicopathological characteristics of patients undergoing curative hepatectomy for HCC.

Clinical variables	NLR-PLR 0	NLR-PLR 1	NLR-PLR 2	*p*
	n = 64 (49%)	n = 32 (24%)	n = 36 (27%)	
*Patient factors*				
Male (n, %)	55 (85.9%)	29 (90.6%)	32 (88.9%)	0.78
Age(years; mean ± SD)	64.5±8.9	67.8±8.8	63.9±12.6	0.24
Hepatitis B (n, %)	39 (60.9.%)	18 (56.2%)	18 (50.0%)	0.57
Child-Pugh Class B (n, %)	1 (1.6)	3 (9.4%)	9 (25.0%)	**<0.01**

*Laboratory data*				
ICG>15 (n, %)	21 (32.8%)	7 (21.9%)	2 (5.6%)	**0.01**
Creatinine (mean ± SD)	104±133	95±43	87±101	0.69

*Surgical factors*				
Major hepatectomy (n, %)	26 (40.6%)	12 (37.5%)	23 (63.9%)	**0.04**
Operation time >350min (n, %)	21 (32.8%)	7 (21.9%)	15 (41.7%)	0.22
EBL >500ml (n, %)	37 (57.8%)	19(59.4%)	25 (69.4%)	0.50

*Tumor factors*				
AFP >400ug/L (n, %)	11 (20.8%)	6 (18.8%)	1 (2.7%)	0.08
Tumor size >5 (n, %)	20 (31.2%)	12 (37.5%)	28(77.8%)	**<0.01**
Multiple tumors (n, %)	12 (18.8%)	10 (31.2%)	8 (22.2%)	0.38
R1 resection (n, %)	4 (6.2%)	2 (6.2%)	7 (19.4%)	0.08

*Postoperative factors*				
Wound infection	2 (3.1%)	3 (9.4%)	1 (2.8%)	0.32
Pneumonia	10 (15.6%)	5 (15.6%)	7 (19.4%)	0.87
UTI	3 (4.7%)	3 (9.4%)	1 (2.8%)	0.46
Bile leak	0 (0)	0 (0)	2 (5.6%)	0.07
Intra-abdominal collection	2 (3.1%)	0 (0)	2 (5.6%)	0.41
Liver failure	4 (6.2%)	3 (9.4%)	4 (11.1%)	0.68

*Follow-up*				
30-day mortality	2 (3.1%)	1 (3.1%)	0 (0)	0.52
LOS (days, mean ± SD)	11.2±11.6	13.4±20.6	19.7± 26.2	0.22
OS (months, median [IQR])	26.5 (11-48)	19.5 (5-36)	24.5 (7-37)	**0.02**
RFS (months, median [IQR])	20 (7-40.5)	12 (5-25)	14.5 (4-28)	**0.02**

NLR: neutrophil-to-lymphocyte ratio; PLR: platelet-to-lymphocyte ratio; ICG: indocyanine green; EBL: estimated blood loss; AFP: a-fetoprotein; UTI: urinary tract infection; LOS: length of stay; OS: overall survival; RFS: recurrence free survival; IQR: interquartile range; SD: standard derivation.

## Data Availability

The data used to support the findings of this study are available from the corresponding author upon request.
